# Adjuvant Vitamin C in critically ill patients undergoing renal replacement therapy: What’s the right dose?

**DOI:** 10.1186/s13054-018-2190-y

**Published:** 2018-11-22

**Authors:** Paul E Marik, Michael H Hooper

**Affiliations:** Division of Pulmonary and Critical Care Medicine, Eastern Virginia Medical School, 825 Fairfax Av, Suite 410, Norfolk, VA 23507 United States of America

**Keywords:** vitamin C, pharmacokinetics, dialysis, continuous renal replacement therapy

Recently there has been increased interest in the use of intravenous vitamin C as adjuvant therapy in patients with sepsis, acute respiratory distress syndrome (ARDS), burns and after cardiac arrest. The optimal dosage strategy in these patients is unclear with dosages varying from 50 mg/kg/day to 1584 mg/kg/day having being studied. The pharmacokinetics of intravenous vitamin C has been studied in healthy volunteers and critically ill patients. In healthy volunteers the pharmacokinetics of intravenous vitamin C are best described by a two-compartment linear dose-response relationship with a 1.25 g dose producing a peak concentration of approximately 885 umol/l [1]. De Grooth et al. studied the pharmacokinetics of a twice daily bolus of 1 g of vitamin C in critically ill septic patients [2]. The mean peak serum vitamin C concentration was 180 umol/l with a trough concentration of between 60 and 80 umol/l; approximately 50% of the administered dose was renally excreted. Vitamin C is freely filtered in the glomerulus with renal losses increasing with increasing plasma concentrations. As a large proportion of the vitamin C dose is renally excreted, serum levels will be significantly higher in anuric patients. Vitamin C is removed by renal replacement therapy [3–5]. The serum levels of vitamin C in patients with acute kidney injury (AKI) receiving adjuvant intravenous vitamin C and who are being treated with continuous renal replacement therapy (CRRT) has not been evaluated. Recently, Honore and colleagues proposed that patients (post cardiac arrest) not being treated with CRRT receive 6 g vitamin C daily and that the dose should be increased to 12 g/day in those receiving CRRT [6]. While we strongly agree that 6 g/day is likely the optimal dose of intravenous vitamin C in critically ill patients, [7] there is no data to suggest that the dose should be increased in patients receiving CRRT. Assuming a plasma vitamin C concentration of 200 umol/l with a hemofiltration dose (CVVH) of 2 L/h and a sieving coefficient of 1, the patient would lose 1.68 g/day (200*2*24 = 9600 umol/day). A daily dose of 6 g/day should adequately cover this loss. As part of an IRB approved study investigating the accuracy of point-of-care glucose monitors (Accu-Chek, Roche, Indianapolis, IN) in septic patients (*n* = 72) receiving intravenous vitamin C (6 g daily), we measured steady state trough and peak (within 30 min of end of infusion) vitamin C levels in 12 patients (Table 1) [8]. While the sample size is small, our limited data does not suggest dosage adjustment is required in patents receiving renal replacement therapy. Additional studies are required to confirm our findings.

**Table 1:** Vitamin C trough and peak levels stratified by renal funcion

	S-creatinine (mg/dl)	Vitamin C -trough (umol/l)	Vitamin C- peak (umol/l)
No AKI (*n* = 5)	0.56 ± 0.25	224 ± 121	543 ± 276
AKI + CRRT (*n* = 4)	1.3 ± 1.1	263 ± 129	461 ± 194
CRF + Intermittent HD (*n* = 3)	4.6 ± 3.1	346 ± 126	914 ± 205

*AKI* acute kidney injury, *CRRT* continuous renal replacement therapy, *CRF* chronic renal failure, *HD* hemodialysis
